# What Do *Pneumocystis* Organisms Tell Us about the Phylogeography of Their Hosts? The Case of the Woodmouse *Apodemus sylvaticus* in Continental Europe and Western Mediterranean Islands

**DOI:** 10.1371/journal.pone.0120839

**Published:** 2015-04-01

**Authors:** Christine Demanche, Manjula Deville, Johan Michaux, Véronique Barriel, Claire Pinçon, Cécile Marie Aliouat-Denis, Muriel Pottier, Christophe Noël, Eric Viscogliosi, El Moukhtar Aliouat, Eduardo Dei-Cas, Serge Morand, Jacques Guillot

**Affiliations:** 1 Laboratoire de Parasitologie (EA4547), Faculté de Pharmacie, Université de Lille, Lille, France; 2 Institut Pasteur de Lille, Centre d’Infection et d’Immunité de Lille, Inserm U1019, UMR CNRS 8204, Université de Lille, BioPôle d'Alfort, Biologie et Diversité des Pathogènes Eucaryotes Emergents, Lille, France; 3 ENVA, UPEC, Research group Dynamyc, Ecole Nationale Vétérinaire d’Alfort, 94704, Maisons-Alfort Cedex, France; 4 CBGP (Centre de Biologie et de Gestion des Populations), UMR INRA/IRD/Cirad/Montpellier SupAgro, Campus international de Baillarguet, CS 30016, 34988, Montferrier-sur-Lez cedex, France; 5 Institut de Botanique (B22), University of Liège, 4000, Liège, (Sart Tilman), Belgium; 6 Muséum national d’histoire naturelle, CR2P—UMR 7207 CNRS, MNHN, Univ Paris06, Paris, France; 7 Departement of Biostatistics (EA2694), Université de Lille, Lille, France; 8 Geneius Laboratories Ltd, INEX Business Centre, Newcastle upon Tyne, United Kingdom; 9 Parasitologie-Mycologie (EA4547) Faculté de Médecine, Université de Lille, CHRU, Lille, France; 10 Institut des Sciences de l’Evolution, UMR CNRS-IRD-UM2, Université de Montpellier 2, F-34093, Montpellier, France; 11 CIRAD-CNRS, Centre d’Infectiologie Christophe Mérieux du Laos, Vientiane, Lao PDR; University of Lausanne, SWITZERLAND

## Abstract

*Pneumocystis* fungi represent a highly diversified biological group with numerous species, which display a strong host-specificity suggesting a long co-speciation process. In the present study, the presence and genetic diversity of *Pneumocystis* organisms was investigated in 203 lung samples from woodmice (*Apodemus sylvaticus*) collected on western continental Europe and Mediterranean islands. The presence of *Pneumocystis* DNA was assessed by nested PCR at both large and small mitochondrial subunit (mtLSU and mtSSU) rRNA loci. Direct sequencing of nested PCR products demonstrated a very high variability among woodmouse-derived *Pneumocystis *organisms with a total number of 30 distinct combined mtLSU and mtSSU sequence types. However, the genetic divergence among these sequence types was very low (up to 3.87%) and the presence of several *Pneumocystis* species within *Apodemus sylvaticus* was considered unlikely. The analysis of the genetic structure of woodmouse-derived *Pneumocystis* revealed two distinct groups. The first one comprised *Pneumocystis* from woodmice collected in continental Spain, France and Balearic islands. The second one included *Pneumocystis* from woodmice collected in continental Italy, Corsica and Sicily. These two genetic groups were in accordance with the two lineages currently described within the host species *Apodemus sylvaticus*. *Pneumocystis* organisms are emerging as powerful tools for phylogeographic studies in mammals.

## Introduction

Phylogeography is a field of research, which studies the principles and processes determining the geographical distribution of genetic lineages. Quite frequently, parasites provide an additional source of information and can better reconstruct the common history of hosts and parasites [1]. For this reason, some parasites have been recently used as phylogeographic markers [1–7]. As an example, host associations of Spinturnicid mites likely result from a complex interaction between the phylogenetic history of the bat hosts and the behaviour and the ecology of both parasites and hosts [6]. Moreover, Spinturnicid mites seem to be a biological tag to reveal the former presence of a nowadays locally extinct bat host species [7]. Similarly, studies on the co-divergence between *A*. *sylvaticus* and the parasitic nematode *Heligmosomoides polygyrus* [2, 8] demonstrated a general association between the parasite genetic structure and the host evolutionary history.

*Pneumocystis* organisms are opportunistic fungi that infect the lungs of a wide range of mammalian species, including humans [9–11]. These atypical fungi are considered to be highly diversified with numerous species [12–14] which display a strong host specificity [10–11, 14–19]. Many molecular studies suggest the existence of a co-speciation process occurring between *Pneumocystis* organisms and their corresponding hosts [10–11, 14, 18, 20–21]. Consistently, in primate-derived *Pneumocystis* organisms, specific DNA sequence divergence among *Pneumocystis* species was correlated with the phylogeny of their specific hosts [10, 21, 22]. Similar observations were noticed at the *Pneumocystis* infra-specific level for the migrating bat *Tadarida brasiliensis* where *Pneumocystis* strain polymorphism could be used as a phylogeographic tool to track natural host populations [18–19].

The woodmouse (*Apodemus sylvaticus*, Rodentia, Muridae) is a common forest dweller, and has been present throughout Europe for at least 3 million years [23]. *Apodemus sylvaticus* is one of the most intensively studied species in the genus. The phylogeographic history of this species in Europe and in the Middle East is well known, proposing an original pattern of postglacial colonization where the Iberian and southern France refuge populations colonized almost all European regions [23–24].

In the present study, we detected and characterised *Pneumocystis* organisms in woodmice from western continental Europe and Mediterranean islands. Questions regarding host and parasite co-divergence as well as comparative phylogeography were specifically addressed. Our objective was to assess whether *Pneumocystis* organisms can be used as phylogeographic markers for rodents, in addition to previous studies on parasites of *Apodemus* and in order to demonstrate that *Pneumocystis* is a promising biological tag for mammalian hosts.

## Materials and Methods

### Ethics statement

**Fig 1:**
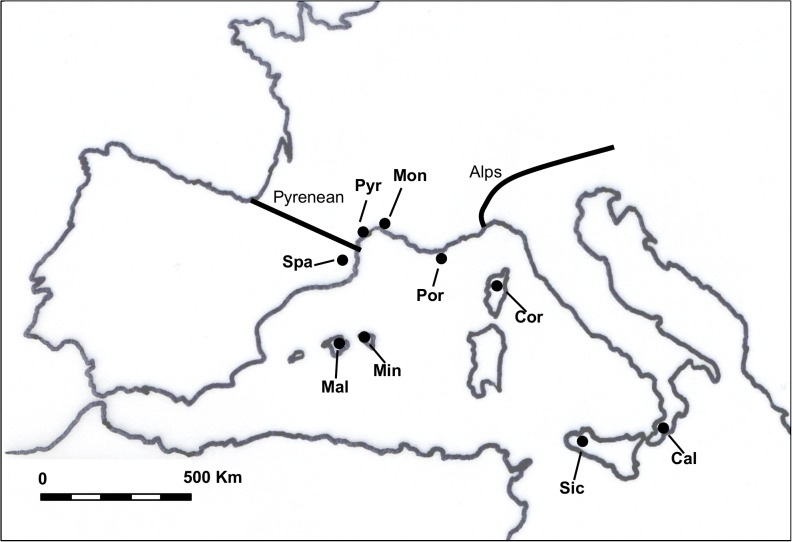
Trapping locations of *Apodemus sylvaticus*. Samples are designated according to the location in which they were collected. The number of examined field mice is indicated between brackets. Spa, Montseny (42); Pyr, the Pyrenees (15); Mon, Montpellier (21); Cal, Calabria (39); Mal, Mallorca (25); Min, Minorca (14); Sic, Sicily (32); Por, Porquerolles (10); Cor, Corsica (5). The thick lines correspond to the main European biogeographic barriers: the Pyrenees (west) and the Alps (east). Sample locations are shown by circles.

All animal experiments were performed according to the directive 2010/63/EEC on the Protection of Animals Used for Experimental and Other Scientific Purposes. The animal work also complied with the French law (nu 2012–10 dated 05/01/2012 and 2013–118 dated 01/02/2013). The rodents, *Apodemus sylvaticus*, were captured using Sherman traps from July until October, 2001. The trapping locations in which woodmice were collected are described in Fig. 1. The study of woodmice did not require to be approved by an ethical committee (European directives 86–609 CEE and 2010/63/EEC). This species is not protected, and no experiment was performed on living animals. No permit approval was needed as this species was trapped outside any preserved areas (national parks or natural reserves). Oral authorization was received from land owners to get trapping authorization on their property (forests or land shrubs). The rodents were euthanized by vertebrate dislocation immediately after capture in agreement with the legislation and the ethical recommendations (2010/63/EEC annexe IV) (see also protocol available on http://www.ceropath.org/references/rodent_protocols_book). All experimental protocols involving animals were carried out by qualified staff (agreement number: 59–350070, accreditation number of the Lille Pasteur Institute: A59107, accreditation number of the Center of Biology and Management of the Populations (CBGP) for wild and inbred animal manipulations: A34-1691). This study is part of a project entitled « Insularité et Parasitisme: diversité parasitaire et écologie évolutive des populations de rongeurs insulaires » supported by the French Institute of Biodiversity.

### Samples

Woodmice were trapped on islands and mainland areas in western Europe (Fig. 1). Mainland areas included Calabria (Italy), Montseny (Spain), Montpellier and the Pyrenees (France). The Mediterranean islands were Mallorca and Minorca (Spain), Corsica and Porquerolles (France), and Sicily (Italy). These Apodemus samples are conserved in the collection of the Conservation Genetics Unit of the University of Liège (currator J.R. Michaux). All organs and viscera were dissected following capture. The lungs were immediately excised and stored at -20°C until analysis. A total number of 203 woodmice lung samples were collected.

### DNA extraction

DNA extraction from lung tissue samples was performed using DNeasy tissue kit (Qiagen, Courtaboeuf, France) following the manufacturer procedure with some modifications. Lysis of lung tissue was done overnight in an incubator at 56°C with permanent rotation. In order to concentrate *Pneumocystis* DNA, the column was re-hydrated with 100 μl of elution AE buffer instead of 200 μl. Extracted DNA was stored at -20°C prior to amplification. A negative control was systematically included in each series of DNA extraction.

### Detection of *Pneumocystis* organisms by nested PCR

*Pneumocystis* DNA was detected in pulmonary samples by nested PCR at the mitochondrial large subunit (mtLSU) rRNA and the mitochondrial small subunit (mtSSU) rRNA genes.

At the mtLSU rRNA locus, the first round of nested PCR consisted of 30 cycles and was performed using the external primer set PH/PE [25]. Each cycle consisted of 3 steps: denaturation for 30s at 94°C, annealing for 1 min at 50°C, and extension for 1 min at 72°C. Five percent (vol/vol) of the amplified product from the first PCR round was used as starting matrix in the second round of PCR. The second PCR round consisted of 30 cycles and was performed using the internal primer set PX/PZ [20]. Each cycle consisted of 3 steps: denaturation for 30s at 94°C, annealing for 1 min at 55°C, and extension for 2 min at 72°C.

At the mtSSU rRNA locus, the first round of nested PCR consisted of 40 cycles and was performed using the external primer set pAZ112-10F-R1 / pAZ112-10R-R1 [26]. Each cycle consisted of 3 steps: denaturation for 30 s at 94°C, annealing for 1 min at 55°C, and extension for 1 min at 65°C. Five percent (vol/vol) of the amplified product from the first round of PCR was used as starting matrix in the second round. The second round of nested PCR consisted of 40 cycles and was performed using the internal primer set pAZ112-13-R1 / pAZ112-14-R1 [26]. Each cycle consisted of 3 steps: denaturation for 30 s at 94°C, annealing for 1 min at 52°C, and extension for 1 min at 65°C for 10 cycles; and denaturation for 30 s at 94°C, annealing for 1 min at 63°C, and extension for 1 min at 65°C for 30 cycles.

Negative controls were included in each experiment, in both DNA extraction and PCR amplification. When non-specific bands were detected, amplification products of expected size (about 250–300 pb) were extracted from a 2% agarose gel (run in Tris-borate-EDTA buffer) using a PCR purification kit (QIAEX II Gel Extraction Kit, Qiagen, Courtabœuf, France). Purified products were sent for sequencing to GenoScreen (Pasteur campus, Genopole of Lille, France). When a unique band of expected size was present, amplified products were directly sent for purification and sequencing to GenoScreen. Sequencing from both ends using sets of internal primers was performed on an automated DNA sequencer (3730XL DNA Analyser, Applied Biosystems). Not all the positive samples were sequenced; representative positive samples were randomly chosen to be sequenced.

### Nucleotide sequence accession numbers

*Pneumocystis* mtLSU rDNA or mtSSU rDNA sequences obtained in this study were deposited in GenBank under accession numbers KF411466, KF384955 to KF385004, and KF384913 to KF384954.

### Statistical analysis

Statistical analyses were performed using SAS (SAS v9, SAS Institute). Variables are described as counts (proportions). A Mc Nemar test was performed to assess the agreement between detection of *Pneumocystis* DNA using mtLSU and mtSSU rRNA. *Pneumocystis* infection was investigated using a two-way analysis of variance with country and insularity as crossed fixed-effect factors, analyzed with a generalized linear mixed model approach (proc GLIMMIX). Models were built with a binomial error distribution and the logit link function. Two-sided p-values<0.05 were deemed to be statistically significant.

### Sequence analysis

The mtLSU and mtSSU rRNA gene sequences described in this study were concatenated and initially aligned with homologous sequences of *Pneumocystis* from the mouse *Mus musculus* (*P*. *murina* GenBank accession numbers AF257179 and KF384954, respectively) and from the squirrel *Sciureus aestuans* (GenBank accession numbers KF411466 and KF384953, respectively) using CLUSTAL X v1.63b [27]. The sequence alignment was then refined by visual optimization using Se-Al v2.0a11 [28] respecting the four criteria defined by Barriel [29]: (1) minimize the number of mutations, which means substitution and insertion-deletion events, and prefer substitution to insertion-deletion; (2) favour transitions to transversions because they are more frequent; (3) minimize the number of sites affected by mutation; (4) minimize the number of phylogenetic implications. All analyses were performed using sequences from *P*. *murina* and *Pneumocystis* of the squirrel *S*. *aestuans* as outgroups. Once the alignment was finalised, the insertion-deletion events were coded as “id” to exploit their phylogenetic information, independently of their size, following the procedure of Barriel [29]. This procedure is particularly useful for complex zones with complex insertions/deletions with substitutions. This strategy requires the introduction of three new character states in the matrix: “i” (insertion), “d” (deletion) and question marks “?” as missing data.

Maximum parsimony (MP) analysis was conducted using Phylogenetic Analysis Using Parcimony Program (PAUP) v4.0b9 [30]. A MP tree was inferred using a heuristic search of 1,000 random additions of sequences with TBR (tree bisection reconnection) branch-swapping. All sites were equally weighed, gaps were scored as missing (the ID code was included in the matrix, so gaps were taken into account).

In a second step, the positions corresponding to missing data in some sequences were removed from the original alignment. Maximum likelihood (ML) analysis of sequences was carried out with PHYML v3.0 [31] using the GTR (general time reversible) + Γ (gamma distribution of rates with four rate categories) + I (proportion of invariant sites) model. The appropriate model of sequence evolution was selected using the program jModelTest2 [32]. Bootstrap values (BVs) were obtained from 1,000 pseudo-replicates. Bayesian analysis was also performed with the program MrBAYES v3.1 [33] on the same data set using the same model and Bayesian Posterior Probabilities (BPPs) were calculated using a Markov Chain Monte Carlo (MCMC) sampling approach [34]. The starting tree was random, and four simultaneous Markov chains were run in duplicates for 2 million generations with burn-in values set at 10,000 generations (based on empirical values of stabilizing likelihoods). Trees were sampled every 100 generations (the other settings being set to default). Distance matrices and bootstrap sampling (1,000 pseudo-replicates) were subsequently generated by using Seqboot and Dnadist combination (Phylip v.3.69) with the Jukes Cantor model. Matrices were analysed using BioNJ [35] and consensus tree computed with Consense (Phylip v.3.69). Alignments of mtLSU and mtSSU rRNA gene sequences utilised in the various analyses are available upon request to the corresponding author.

### Phylogeographic analyses

A minimum spanning network was constructed using the MINSPNET algorithm available in the ARLEQUIN 2.0 program [36]. The genetic divergences (GD) between sample groups were estimated using a distance analysis (K_2_P, mega program). The nucleotide (π) and haplotype (h) diversities were estimated using the DNA sequence polymorphism (DnaSP) version 5 program [37].

## Results

### Detection of *Pneumocystis* DNA in woodmice lungs

A sample was considered to be positive for the presence of *Pneumocystis* DNA when a specific band was amplified by PCR at the expected size either at the mtLSU or mtSSU rRNA locus or at both loci (Table 1). *Pneumocystis* DNA was detected in 189 out of 203 woodmice lung samples examined (93.1%). Amplifications at both mtSSU and mtLSU rDNA were positive for the same individual in 38 animals (Table 1).

**Table 1:** Prevalence of *Pneumocystis* carriage in woodmice (*Apodemus sylvaticus*) according to rodent geographic origin.

Countries	Regions	Positive^1^ *Pneumocystis* samples / total sample number (%)	Targeted loci sequenced LSU (distinct seq. type)	Targeted loci sequenced SSU (distinct seq. type)	Reference of concatenated sequences (LSU +SSU) of positive *Pneumocystis* from individual samples (see Figs. 1–3)
Italy	Sicily (Sc)	26/32 (81.2%)	12 (11)	8 (5)	Sc3; Sc5; Sc8; Sc9; Sc12; Sc13; Sc 14; Sc16
Italy	Calabria (Cal)	38/39 (99.4%)	4 (4)	5 (3)	Cal6; Cal7; Cal8; Cal39^a^
Spain	Montseny (Spa)	41/42 (97.6%)	8 (5)	5 (2)	Spa3; Spa4; Spa5; Spa15; Spa19^b^
Spain	Minorca (Mn)	12/14 (85.7%)	4 (3)	2 (1)	Mn33; Mn58
Spain	Mallorca (Ma)	24/25 (96.0%)	7 (2)	8 (2)	Ma47^c^; Ma51^c^; Ma53^d^; Ma59^c^; Ma62^d^; Ma66^d^; Ma71^d^
France	Montpellier (Mon)	21/21 (100%)	6 (5)	5 (2)	Mon4; Mon5^b^; Mon6; Mon12; Mon23
France	Pyrenean (Pyr)	14/15 (93.3%)	5 (3)	3 (1)	Pyr1; Pyr3; Pyr14
France	Porquerolles (Por)	9/10 (90.0%)	1 (1)	1 (1)	Por15^b^
France	Corsica (Cor)	4/5 (80.0%)	3 (3)	3 (2)	Cor1; Cor2; Cor5^a^
Total in all areas		189/203 (93.1%)	50 (32)	40 (13)	38 concatenated sequences of which 30 are variable

Woodmice lung DNA samples were screened by nested-PCR at both mtLSU rRNA (LSU) and mtSSU rRNA (SSU) loci for the presence of *Pneumocystis*. The number of woodmice lung samples positive^1^ for *Pneumocystis* DNA is indicated over the total number of analysed samples. Results of *Pneumocystis* carriage in wood mice are given according to woodmice geographic origin.

^1^ A wood mice lung sample is considered to be positive for *Pneumocystis* DNA if the nested-PCR reveals to be positive at either mtLSU rRNA or mtSSU rRNA or both loci.

^a^ *Pneumocystis* concatenated sequences from the samples Cal39 and Cor5 are the same

^b^ *Pneumocystis* concatenated sequences from the samples Spa19, Mon5 and Por15 are the same

^c^ *Pneumocystis* concatenated sequences from the samples Ma47, Ma51 and Ma59 are the same

^d^ *Pneumocystis* concatenated sequences from the samples Ma53, Ma62, Ma66 and Ma71 are the same

### Potential influence of country and insularity *versus* mainland in *Pneumocystis* carriage

No significant statistical association could be established between the presence of *Pneumocystis* DNA and the country of origin of animals (p = 0.79) (Table 2). No interaction was observed between country and insularity (p = 0.85). However, the frequency of *Pneumocystis* DNA detection was significantly higher in woodmice from continent than in insular woodmice (p = 0.02). The probability of picking a *Pneumocystis*-infected woodmouse was 6-fold higher on the continent than on islands (Table 2).

**Table 2:** *Pneumocystis* carriage in woodmouse: influence of the country of origin and insularity *versus* mainland (multivariate analysis)^a^.

Variable	OR	CI95%	p
Country			0.79
France	1		
Spain	1.471	[0.260; 8.304]	
Italy	0.851	[0.158 ; 4.592]	
Insularity			0.02
Yes	1		
No	5.444	[1.382; 21.449]	
Interaction country x Insularity	/	/	0.85

^a^ OR, odds ratio; CI, confidence interval. Significant results at *P* = 0.05 country × insularity

### Sequence analysis

Direct sequencing of nested PCR products demonstrated a very high variability among woodmice-derived *Pneumocystis* sequences. A total number of 32 distinct mtLSU sequence types and 13 distinct mtSSU sequence types were detected. Sequences at the mtLSU rRNA locus seemed to be more variable than those at the mtSSU rRNA locus (Table 1). After combining mtLSU and mtSSU sequences, 30 distinct sequence types were generated (Tables 1 and 3). Phylogenetic analyses were then carried out using the concatenated sequence dataset. A total number of 555 bp (360 bp and 195 bp from mtSSUrRNA and mtLSUrRNA loci, respectively) was selected in the subsequent phylogenetic analyses. The initial alignment included 32 taxa (2 outgroups and 30 taxa ingroups), 555 characters and 513 common sites. After coding the insertion-deletion events [29], the final alignment with the ID code included 32 taxa and 584 characters.

**Table 3:** Net and total genetic divergence existing among the *Pneumocystis* concatenated mtLSU and mtSSU sequences isolated from woodmice captured in various geographic localities.

	% of net genetic divergence (% of total genetic divergence) from							
Woodmice sample localities	1. Sc	2. Cal	3. Spa	4. Mn	5. Ma	6. Mon	7. Pyr	8. Por
1. Sicily								
2. Calabria	0.2 (0.9)							
3. Montseny (Spain)	**1.2 (1.8)**	**1.0 (1.5)**						
4. Minorca	**1.0 (1.8)**	**0.8 (1.6)**	0.2 (0.5)					
5. Mallorca	**1.3 (1.7)**	**1.1 (1.4)**	0.0 (0.3)	0.1 (0.4)				
6. Montpellier	**1.4 (2.5)**	**1.3 (2.3)**	0.1 (1.1)	0.0 (1.1)	0.1 (0.9)			
7. Pyrenees	**1.2 (2.1)**	**1.0 (1.8)**	0.1 (0.8)	0.2 (0.8)	0.0 (0.7)	0.2 (1.4)		
8. Porquerolles	**1.4 (1.8)**	**1.4 (1.7)**	0.1 (0.4)	0.0 (0.4)	0.2 (0.3)	0.2 (0.9)	0.2 (0.8)	
9. Corsica	0.3 (0.8)	0.0 (0.5)	**1.1 (1.5)**	**0.9 (1.5)**	**1.2 (1.4)**	**1.4 (2.2)**	**1.1 (1.8)**	**1.4 (1.5)**

High percentages of divergence (bold text) are found when comparing *Pneumocystis* sequences from italo-corsican and western-european (Spain, France, Balearic islands) woodmice.

### Genetic diversity

**Fig 2:**
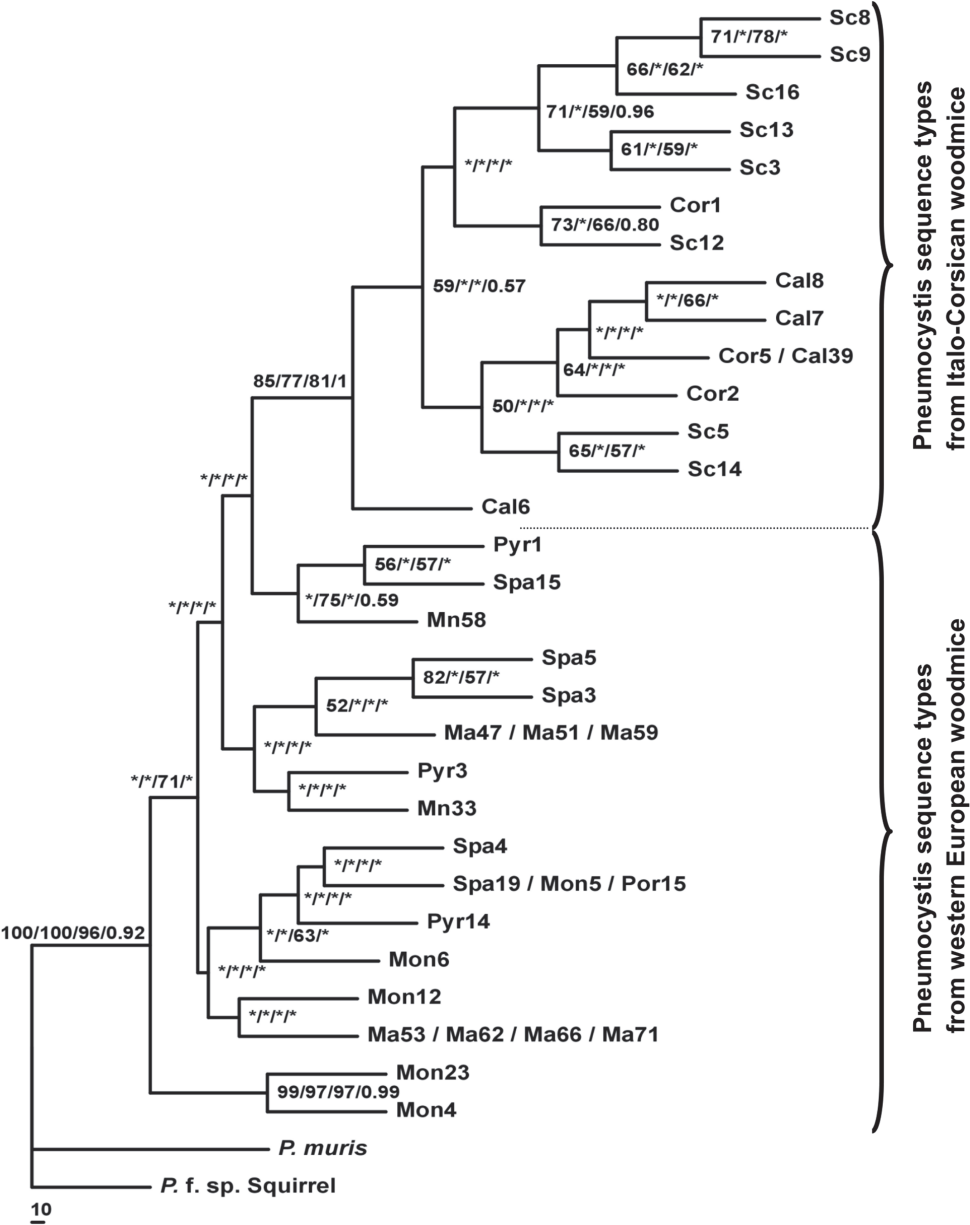
Phylogenetic tree depicting relationships between *Pneumocystis* organisms isolated from *A*. *sylvaticus* of different trapping locations. NJ, MP, ML and MB are inferred from the combined mitochondrial small subunit (mtSSU) and large subunit (mtLSU) rRNA sequences. The percentages displayed above the branches are the frequencies with which a given branch appeared in 1,000 bootstrap replications in NJ/MP/ML/MB analyses respectively. *Bootstrap values below 50% are depicted by a star.

Most of the combined sequences corresponded to specific geographical areas (Tables 1; Fig. 2). Three combined *Pneumocystis* sequence types were detected in the woodmice from the Pyrenees (France). Similarly, unique *Pneumocystis* sequence types were characterized in the woodmice captured in the Spanish islands of Mallorca (2 sequence types) and Minorca (2 sequence types). As for Sicilian woodmice, 7 distinct *Pneumocystis* sequence types were detected. For Montpellier (France), we detected 4 specific *Pneumocystis* sequence types; another sequence type (Mon5, Table 1) was common with 2 other ones isolated in woodmice from Montseny (Spain, Spa19) and Porquerolles (France, Por15). Among the 3 specific *Pneumocystis* sequence-types found in the woodmice from Calabria (Italy), one *Pneumocystis* sequence was common with that found in a woodmouse from Corsica (France). In addition, 2 unique *Pneumocystis* sequence-types were detected in the corsican woodmice (Table 1).

The total genetic divergence among the studied localities (Table 3) delineated 2 groups of combined sequence types. The first one, named the Western European clade, comprised *Pneumocystis* from woodmice collected in continental Spain (Montseny), France and Balearic islands (range of genetic divergence: 0% to 0.2%). The second one, named the Italo-Corsican clade, included *Pneumocystis* from woodmice collected in continental Italy (Calabria), Corsica and Sicily (range of genetic divergence: 0% to 0.3%). The genetic divergence existing between the Western-European and Italo-Corsican clades ranged from 0.8% to 1.4% and is higher than the genetic divergence existing within each clade (Table 3).

**Fig 3:**
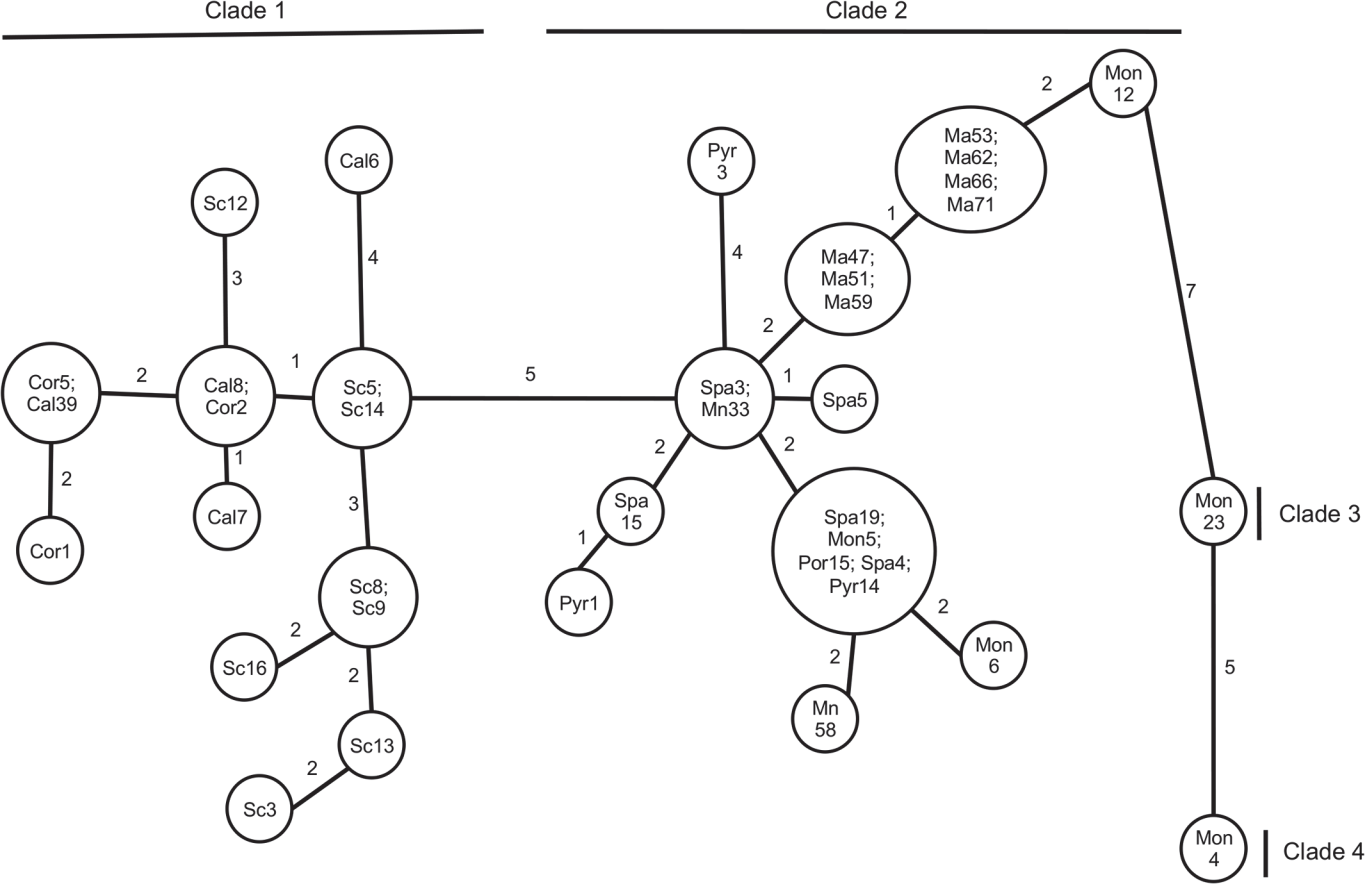
Minimum spanning network constructed using mtLSU rRNA and mtSSU rRNA combined sequences of *Pneumocystis* from *A*. *sylvaticus*. Geographic origins (see Table 1 and Fig. 1) are indicated: Spa, Montseny; Pyr, the Pyrenees; Mon, Montpellier; Cal, Calabria; Mal, Mallorca; Min, Minorca; Sic, Sicily; Por, Porquerolles; Cor, Corsica. Numbers correspond to the mutational steps observed between haplotypes.

When comparing the genetic diversity values of *Pneumocystis* isolated from woodmice captured on islands *versus* those from host populations originating from mainland, a trend toward the loss of *Pneumocystis* genetic diversity was noticed. Indeed, the nucleotide diversity tends to decrease (π) in the *Pneumocystis* sequences isolated from insular host populations except for Sicily (8 individuals and 8 sequences types) and Minorca (2 individuals and 2 sequences types) (Table 4). With regard to both *Pneumocystis* groups (Fig. 2 and 3), the sole insular *Pneumocystis* population that seemed to display levels of genetic divergence (GD) and mean nucleotide diversity (π) that were comparable to those assessed in population from continental Italy (Calabria) was isolated from Sicilian rodents. All other *Pneumocystis* populations from Mediterranean islands were characterised by lower values of GD and π than mainland source populations except for Minorca (Table 4).

**Table 4:** Genetic variability of *Pneumocystis* concatenated mtLSU and mtSSU sequences observed within each host locality.

Locality	Sample size	Number of combined sequence types	GD (%)	π (SD)	h (SD)
Calabria (Cal)	4	4	0.6	0.0086 (0.0029)	1 (0.177)
Corsica (Cor)	3	3	0.2	0.0051 (0.0017)	1 (0.272)
Sicily (Sc)	8	6	0.7	0.0074 (0.0011)	0.929 (0.084)
Montseny (Spa)	5	4	0.5	0.0047 (0.0011)	0.900 (0.161)
Montpellier (Mon)	5	5	1.4	0.0138 (0.0030)	1 (0.126)
Pyrenean (Pyr)	3	3	1.1	0.0102 (0.0028)	1 (0.272)
Mallorca (Ma)	7	2	0.1	0.0011 (0.0002)	0.571 (0.119)
Minorca (Mn)	2	2	0.8	0.0077 (0.0038)	1 (0.500)

GD, genetic divergence; π, nucleotidic diversity; h, haplotype diversity; SD, standard deviation

The *Pneumocystis* continental populations from Calabria, Montpellier and the Pyrenees displayed the highest levels of π except for Montseny (Table 4).

### Phylogenetic analyses

The matrix of 584 characters included 440 constant characters, 95 variable parsimony-uninformative characters and 49 parsimony-informative characters. The analysis yielded 31 738 equally parsimonious trees (length = 213 steps, CI = 0.761 and RI = 0.748).

The analysis of the genetic structure of woodmice-derived *Pneumocystis* revealed two distinct groups (Fig. 2). The *Pneumocystis* clade from Italy and Corsica was well individualized in the phylogenetic analyses (NJ, MP, ML, MB) and it was supported by bootstrap values higher than 80%. A small subgroup corresponding to most of the *Pneumocystis* sequences isolated from woodmice trapped in Sicily (Sc3, Sc8, Sc9, Sc13 and Sc16), was weakly or well supported by bootstrap values depending on the various analyses applied (Fig. 2).

*Pneumocystis* sequence types from Western European woodmice (Porquerolles and mainland France, Balearic Islands and mainland Spain) always met outside of the Italo-Corsican clade and consequently constituted a second group of *Pneumocystis* that was not well supported by bootstrap values and was not monophyletic (Fig. 2). The relationships existing inside this group remain unclear with the exception of 2 *Pneumocystis* sequence types isolated from 2 woodmice trapped in Montpellier (Mon23 and Mon4) (Fig. 2).

### Phylogeographic analyses

The minimum spanning network displayed 24 haplotypes of *Pneumocystis* clearly distributed in 2 major clades defined above and separated from each other by a genetic distance of 5 mutational steps (Fig. 3). However, two other clades seemed to appear, each constituted by only one *Pneumocystis* haplotype isolated from 2 woodmice trapped in Montpellier (Mon23 and Mon4). These *Pneumocystis* types were clearly divergent from the others. For the 2 major clades, the first one comprised *Pneumocystis* haplotypes resulting from woodmice from Sicily, Calabria and Corsica (clade 1). The second group included *Pneumocystis* haplotypes from woodmice from Spain, Montpelier, Mallorca and Minorca as well as the Pyrenees (Western European clade 2). Within clade 2, almost all haplotypes were connected together in a star-like topology. *Pneumocystis* isolated from woodmice captured in Spain, Montpellier, Porquerolles and the Pyrenees (Spa19, Mon5, Por15, Spa4 and Pyr14) were grouped into the same haplotype. Another *Pneumocystis* sequence-type isolated from woodmice sampled in Spain fell into another haplotype shared with a sample from Minorca (Spa3 and Mn33).

The topology of the Italo-Corsican clade (clade 1) is more heterogeneous than in the Western European clade. We note the proximity between the *Pneumocystis* from the woodmice from Corsica, Calabria and Sicily, but also 2 haplotypes shared by *Pneumocystis* from Calabrian and Corsican woodmice (Fig. 3). On the other hand, Sicilian woodmice possessed their own *Pneumocystis* haplotypes and did not share them with the Corsican or Calabrian woodmice (Fig. 3).

## Discussion

### Prevalence of *Pneumocystis* in *Apodemus sylvaticus*

Previous studies using nested PCR reported important differences in *Pneumocystis* prevalence between mammal species of the same zoological group such as primates or rodents. Prevalence of *Pneumocystis* reached 26.5% in captive primates [38] and 33.6% in healthy macaques maintained in partial release [39]. In micromammals collected in France, *Pneumocystis* prevalence reached 68.0% when molecular techniques were applied [40]. In Thailand, 57.7% of wild rats harboured *Pneumocystis* [41]. *Pneumocystis* was found in rodents of the genus *Apodemus* in several European and Japanese regions, with rates of prevalence ranging from 0 to 43% [42].

The prevalence reported in the present study (93.1%) was much higher than previously cited values. This result may be explained both by the efficiency of molecular amplification used for *Pneumocystis* detection in our study, and by the gregarious behaviour of *A*. *sylvaticus* [43] as crowding favours the transmission of *Pneumocystis* by aerial route [19, 39, 44]. The *Pneumocystis* transmission is influenced by the interactions between mammals and the structuring of mammalian society [19, 39]. Consequently, *Pneumocystis* can be considered as a marker of social structuring of mammals.

### Potential influence of insularity *versus* continent

The prevalence of *Pneumocystis* colonization was significantly higher in woodmice from continent than in the ones from islands. This finding is not in accordance with previous study [45]. Indeed, the prevalence and intensity of parasitism are often higher in insular than in mainland populations for parasites that are directly transmitted from host-to-host. This high insularity prevalence is in relation with the low parasite species richness in insular host populations. A study found that there was a significant influence of insularity on the decrease in parasite species richness of *A*. *sylvaticus* [46], which can be explained by the decrease of mammal diversity [47].

Another study on *H*. *polygyrus* and its host *A*. *sylvaticus* highlighted the “island syndrome” in this parasitic nematode species with a loss of genetic diversity following territory colonization [48]. Our results tend to support this observation (Table 4). In the first clade (Western European *Pneumocystis* woodmice), the Majorcan *Pneumocystis* population displayed a significant loss of genetic diversity (GD = 0.1%; π = 0.0011). Even if nucleotidic diversity of the Minorcan *Pneumocystis* population appeared to be higher than the diversity of the Spanish population (mainland origin), both were yet lower than those of Montpellier and the Pyrenees. Moreover, Nieberding et al. [48] observed in the case of Minorcan *H*. *polygyrus* that the GD and π values were intermediate when compared to those from Spain and Majorcan populations. The lower values of GD and π of *Pneumocystis* Spanish populations seem to reflect past genetic bottlenecks or recent population expansion from a small number of founder individuals.

In the case of the second clade (Calabria, Sicily and Corsica), we observed the same tendency of decrease in *Pneumocystis* genetic and nucleotidic diversity in islands, except for Sicily, thus confirming the results obtained previously for *A*. *sylvaticus* and its nematode parasite *H*. *polygyrus* [23, 48].

The results of the present work support the hypothesis that the *Pneumocystis* Balearic populations originated from South-Western Europe and the Italian region was the continental source for *Pneumocystis* Sicilian and Corsican populations as well as for its host *A*. *sylvaticus* and other parasites like *H*. *polygyrus* [48].

### Genetic diversity of *Pneumocystis* from *Apodemus sylvaticus*

Several studies reported *Pneumocystis* sequence types from various host species. The genetic divergence ranged from 7.5% to 28.2% according to the used locus, i.e. mtLSU rRNA or mtSSU rRNA, and the host species examined [10, 13, 19]. The genetic divergence among *Pneumocystis* organisms from *A*. *sylvaticus* was very low (lower than 3.87% of divergence) and strongly suggested that a single *Pneumocystis* species is present in the woodmouse. The phylogenetic analysis of *Pneumocystis* sequences was in accordance with the host specificity concept of *Pneumocystis* [14–15, 17]. A first investigation about primate-derived *Pneumocystis* organisms demonstrated that *Pneumocystis* phylogeny mirrors its host phylogeny, suggesting long-range functional and genetic adaptation process leading to cospeciation [21]. *Pneumocystis* organisms may have evolved together with their hosts. The low genetic divergence in woodmice-derived *Pneumocystis* organisms is in connection with the geographical place where the rodents were captured.

A phylogenetic tree for *A*. *sylvaticus* was first provided by Michaux et al. [23, 49–50]. According to this analysis, two groups of populations can be distinguished: a first group comprises woodmice from France, Spain as well as Balearic Islands (Western European group); the second group includes woodmice from the Balkans, Italy and Sicily. The phylogenetic study inferred from *Pneumocystis* sequences detected in woodmice showed the same tendency of grouping (Fig. 2). Particularly, *Pneumocystis* from woodmice in Italy, Corsica and Sicily were clustered in the same monophyletic group. In contrast, the other Western European populations of *Pneumocystis* appeared less structured. Concerning the Mediterranean islands, our results pointed out a close relationship of the Porquerolles and Mallorca islands with the continental French and Spanish *Pneumocystis* populations. In contrast, Corsican *Pneumocystis* appeared strongly associated to the Italian and Sicilian *Pneumocystis* populations (Fig. 2 and 3). Michaux et al. [50] demonstrated that *A*. *sylvaticus* underwent a significant bottleneck during Quaternary glaciations, finding refugia in the Iberian Peninsula and the Italo-Balkan areas. While the Iberian populations later spread throughout the rest of Europe, the Italo-Balkan populations remained trapped by surrounding mountains and did not contribute to the genetic constitution of central and north European populations. The results of the study suggest co-divergence: the population structure of woodmice-related *Pneumocystis* is in agreement with the evolutionary history of *A*. *sylvaticus*. *Pneumocystis* organisms were likely carried by their specific host during their migration and evolution.

In a recent study, using a nematode parasite as phylogeographic marker, Callejon et al. [51] underlined two clear-cut geographical and genetic lineages of the nematode *Trichuris muris* isolated from different Muridae hosts, including *A*. *sylvaticus*, in Europe: one lineage was widespread from Northern Spain (Catalonia) to Denmark (Western European region), while the second one colonized the Eastern European region (Croatia, Rumania, and Turkey) [51]. The geographic repartition of *Pneumocystis* sequences is thus in accordance with the historical processes of *Apodemus* speciation described and emphasized by the parasite population evolution.

A focus on the last clade, comprising woodmice from the Balkans, Italy and Sicily, pointed out a division into two subclades showing a large and robust differentiation [23]. Animals from Sicily displayed a higher level of nucleotide diversity than animals from Balkans and continental Italy [23]. Nieberding et al. [8] established the phylogeographical pattern of the European nematode *H*. *polygyrus*, and, like its woodmouse host, the Italo-Balkan lineage comprised a Sicilian subclade. Moreover, Nieberding et al. [48] reported that the southern Italian region is the continental source for Sicilian populations of *H*. *polygyrus* as well as for its host *A*. *sylvaticus*. In the present study, the phylogenetic tree and the minimum spanning network showed a comparable trend to the grouping of woodmice-related *Pneumocystis* in this Sicilian clade, even if some relations remain unclear. Sicilian *Pneumocystis* population displayed levels of GD and π that were comparable to Calabrian source populations. Moreover, many more haplotypes were found in Sicilian *Pneumocystis* than fungi from other regions as depicted by the network analysis. This suggests a more heterogeneous distribution of Sicilian *Pneumocystis* and thus, a more stable population less subjected to a recent expansion. These results tend to confirm the hypothesis in which Sicily would be a ‘hotspot’ of genetic diversity for woodmice and its associated parasites [48].

### *Pneumocystis* spp as proxies

*Pneumocystis* is widely present in ecosystems [12]. Surveys in domestic, synanthropic or wild mammalian species showed that mammals harbor one or several host-specific *Pneumocystis* species or strains [10–11, 14, 18–19, 21, 41]. Similarly, woodmice harbor many host-specific *Pneumocystis* strains apparently related with host infra-specific variants. In addition, *Pneumocystis* polymorphism seems to be related with woodmice geographical distribution.

Some uncertainties remain in the *Pneumocystis* life cycle. Particularly, a question is unanswered: are *Pneumocystis* organisms able to survive or multiply in the environment? A recent study reports that the social or behavioural factors may influence transmission of *Pneumocystis* within the colonies of bat species, while the environmental factors do not seem to have an impact on the carriage rate of *Pneumocystis* [11]. The strong host-species specificity and co-divergence do not support the hypothesis of an environmental reservoir [2, 6, 10–11, 13]. This rather suggests that *Pneumocystis* may only survive very ephemerally in the environment, and that the infection travels from host-to-host via the airborne route, while the crowding of mammals favours fungal transmission. *Pneumocystis* could be used as a tool to study the social structuring and phylogeny of their hosts.

*Pneumocystis* organisms are not only specific of hosts [2–3, 5–6, 8, 52–54], but they co-diverge with their hosts [2–3, 13] and it differs within the same host species according to the geographical place [10–11].

This double feature of *Pneumocystis* organisms, host and site specificities, constitutes a promising tool to unveil the phylogeny or the phylogeography for many mammalian hosts.

### Conclusion

*Pneumocystis* species seem to be good candidates for phylogenetic and phylogeographic studies in *Apodemus sylvaticus*. Indeed, the prevalence of *Pneumocystis* carriage in *A*. *sylvaticus* is higher (93.1%) than that reported for other parasites like the nematode *Heligmosomoides polygyrus* also studied in *A*. *sylvaticus*. Thus, *Pneumocystis* organisms constitute an easily accessible marker. Furthermore, we determined host and parasite co-divergence. In woodmice-derived *Pneumocystis*, specific DNA sequence divergence among *Pneumocystis* genotypes was correlated with the geographic location of their specific hosts. The analysis of *Pneumocystis* genetic diversity confirmed the evolutionary history of *A*. *sylvaticus* in Europe. Furthermore, Sicily was confirmed to be a hotspot of intraspecific biodiversity not only at the level of the hosts but also of their parasites. *Pneumocystis* species and genotypes are emerging as powerful tools to conduct phylogeographic studies in mammals.

## Supporting Information

S1 Table Divergence matrix of *Pneumocystis* combined mtLSU rDNA and mtSSU rDNA sequences amplified from 30 woodmice, two rodents (*P*. *murina* from mouse *Mus musculus* and *Pneumocystis* from squirrel *Sciureus aestuans*). (DOCX)

S1 Fig Alignement of the *Pneumocystis* combined mtSSU rDNA and mtLSU rDNA sequences amplified from 30 woodmice, two other rodents (*P*. *murina* from mouse *Mus musculus* and *Pneumocystis* from squirrel *Sciureus aestuans*). (TXT)

S2 Fig NJ tree depicting relationships between *Pneumocystis* organisms isolated from *A*. *sylvaticus* of different trapping locations. NJ is inferred from the combined mitochondrial small subunit (mtSSU) and large subunit (mtLSU) rRNA sequences. The percentages displayed above the branches are the frequencies with which a given branch appeared in 1,000 bootstrap replications.
(PDF)

S3 Fig ML phylogenetic tree depicting relationships between *Pneumocystis* organisms isolated from *A*. *sylvaticus* of different trapping locations. ML is inferred from the combined mitochondrial small subunit (mtSSU) and large subunit (mtLSU) rRNA sequences. The percentages displayed above the branches are the frequencies with which a given branch appeared in 1,000 bootstrap replications.
(PDF)

S4 Fig MB phylogenetic tree depicting relationships between *Pneumocystis* organisms isolated from *A*. *sylvaticus* of different trapping locations. MB are inferred from the combined mitochondrial small subunit (mtSSU) and large subunit (mtLSU) rRNA sequences. The percentages displayed above the branches are the frequencies with which a given branch appeared in 1,000 bootstrap replications.
(PDF)

S5 Fig MP phylogenetic tree depicting relationships between *Pneumocystis* organisms isolated from *A*. *sylvaticus* of different trapping locations. MP is inferred from the combined mitochondrial small subunit (mtSSU) and large subunit (mtLSU) rRNA sequences. The percentages displayed above the branches are the frequencies with which a given branch appeared in 1,000 bootstrap replications. *Bootstrap values below 50% are reported.
(PDF)
