# Correction: What Do *Pneumocystis* Organisms Tell Us about the Phylogeography of Their Hosts? The Case of the Woodmouse *Apodemus sylvaticus* in Continental Europe and Western Mediterranean Islands

**DOI:** 10.1371/journal.pone.0171282

**Published:** 2017-02-09

**Authors:** Christine Demanche, Manjula Deville, Johan Michaux, Véronique Barriel, Claire Pinçon, Cécile Marie Aliouat-Denis, Muriel Pottier, Christophe Noël, Eric Viscogliosi, El Moukhtar Aliouat, Eduardo Dei-Cas, Serge Morand, Jacques Guillot

Upon re-examination of the samples included in this study, the authors have established that Cal6 is *Apodemus flavicollis* and not *Apodemus sylvaticus* as originally reported [1].

The identification of the *Apodemus* mice samples was completed following the protocol described in Michaux *et al*. [2] based on the sequencing of a mitochondrial cytochrome b fragment. All samples included in this study have been checked using this method.

The sequence obtained from sample Cal6 is in accordance with *Apodemus flavicollis*. This single result does not affect the general conclusions of the article. A recent study showed that *Apodemus flavicollis* and *Apodemus sylvaticus* may harbor the same Pneumocystis type [3]. These *Apodemus* species are closely related ecologically and phylogenetically. They can be trapped together and also show a high level of sympatry. They are members of the same subgenus Sylvaemus [4]. Furthermore, this result could be also explained by the fact that the densities of *A*. *flavicollis* can be more important in these regions with regard to *A*. *sylvaticus*. This would favor the spill-over of Pneumocystis between the two *Apodemus* species in these areas. Such a spill-over, which can lead to host switch, was already observed in the study of Danesi et al. [3], which also supported a host-specificity of Pneumocystis at least at the genus level.

We are including with this Correction a table listing the *Apodemus* individuals with details on their identification (Table 1). The amplified sequence for Cal6 has been deposited in the European Nucleotide Archive under accession number: LT622178, http://www.ebi.ac.uk/ena/data/view/LT622178.

**Table 1:** List of *Apodemus* individuals with ID references, locality, sex, month and year of trapping, season of trapping, weigh (in g) and age. Species determination were based on morphology and genetic markers [5], [6], [7].

ID Current study	ID Goüy de Bellocq (PhD Thesis 2003) [6]	ID Field	Locality	Species*	Reference for Species ID	Sex	Month/ Year	Season	Weight (g)	Age
Cal8	C8	01 10 08 07	Calabria	*A*. *sylvaticus*	[6], [7]	M	10/2001	Autumn	16.5	4
Cal39	C39	01 10 10 09	Calabria	*A*. *sylvaticus*	[6], [7]	F	10/2001	Autumn	16.0	2
Cal7	C7	01 10 10 17	Calabria	*A*. *sylvaticus*	[6], [7]	M	10/2001	Autumn	21.5	3
Cal6	C6	01 10 10 05	Calabria	*A*. *flavicolis*	Current study	-	10/2001	Autumn	-	-
Mon23	M23	01 05 23 04	Montpellier	*A*. *sylvaticus*	[6], [7]	F	05/2001	Spring	20.0	4
Mon6	M6	01 05 22 05	Montpellier	*A*. *sylvaticus*	[6], [7]	M	05/2001	Spring	29.0	4
Mon4	M4	01 05 21 04	Montpellier	*A*. *sylvaticus*	[6], [7]	F	05/2001	Spring	22.0	-
Mon12	M12	01 05 23 07	Montpellier	*A*. *sylvaticus*	[6], [7]	M	05/2001	Spring	30.0	4
Mon5	M5	01 05 21 05	Montpellier	*A*. *sylvaticus*	[6], [7]	M	05/2001	Spring	21.0	-
Pyr1	F1	00 12 01 01	Pyrénées	*A*. *sylvaticus*	[6], [7]	-	12/2000	Winter	-	-
Pyr3	F3	00 12 01 03	Pyrénées	*A*. *sylvaticus*	[6], [7]	-	12/2000	Winter	-	-
Pyr14	F14	00 11 30 08	Pyrénées	*A*. *sylvaticus*	[6], [7]	-	12/2000	Winter	-	-
Spa19	E19	01 09 25 07	Montseny	*A*. *sylvaticus*	[6], [7]	M	10/2001	Autumn	27.0	3
Spa4	E4	01 09 26 01	Montseny	*A*. *sylvaticus*	[6], [7]	M	10/2001	Autumn	24.0	3
Spa5	E5	01 10 18 01	Montseny	*A*. *sylvaticus*	[6], [7]	M	10/2001	Autumn	27.0	3
Spa3	E3	01 09 25 09	Montseny	*A*. *sylvaticus*	[6], [7]	-	10/2001	Autumn	-	-
Spa15	E15	01 09 24 01	Montseny	*A*. *sylvaticus*	[6], [7]	-	10/2001	Autumn	-	-
Por15	M15	01 04 23 04	Porquerolles	*A*. *sylvaticus*	[6], [7]	M	05/2001	Spring	26.0	2
Sc3	S3	01 10 12 02	Sicily	*A*. *sylvaticus*	[6], [7]	F	10/2001	Autumn	26.0	3
Sc9	S9	01 10 11 12	Sicily	*A*. *sylvaticus*	[6], [7]	F	10/2001	Autumn	25.0	4
Sc8	S8	01 10 11 06	Sicily	*A*. *sylvaticus*	[6], [7]	F	10/2001	Autumn	30.5	-
Sc14	S14	01 10 11 05	Sicily	*A*. *sylvaticus*	[6], [7]	F	10/2001	Autumn	17.0	4
Sc12	S12	01 10 09 15	Sicily	*A*. *sylvaticus*	[6], [7]	F	10/2001	Autumn	22.0	4
Sc5	S5	01 10 11 02	Sicily	*A*. *sylvaticus*	[6], [7]	M	10/2001	Autumn	27.5	3
Sc16	S16	01 10 11 03	Sicily	*A*. *sylvaticus*	[6], [7]	F	10/2001	Autumn	10.0	2
Sc13	S13	01 10 09 08	Sicily	*A*. *sylvaticus*	[6], [7]	F	10/2001	Autumn	25.0	4
Ma51	M51	01 07 18 01	Mallorca	*A*. *sylvaticus*	[6], [7]	M	07/2001	Summer	16.0	-
Ma66	M66	01 07 13 01	Mallorca	*A*. *sylvaticus*	[6], [7]	M	07/2001	Summer	19.0	-
Ma47	M47	01 07 19 03	Mallorca	*A*. *sylvaticus*	[6], [7]	F	07/2001	Summer	18.0	3
Ma59	M59	01 07 18 05	Mallorca	*A*. *sylvaticus*	[6], [7]	M	07/2001	Summer	19.0	3
Ma53	M53	01 07 18 03	Mallorca	*A*. *sylvaticus*	[6], [7]	M	07/2001	Summer	27.0	-
Ma62	M62	01 07 20 04	Mallorca	*A*. *sylvaticus*	[6], [7]	F	07/2001	Summer	20.0	3
Ma71	M71	-	Mallorca	*A*. *sylvaticus*	[6], [7]	-	-	-	-	-
Mn58	ME58	01 07 26 04	Menorca	*A*. *sylvaticus*	[6], [7]	M	07/2001	Summer	29.5	4
Mn33	ME33	01 07 25 01	Menorca	*A*. *sylvaticus*	[6], [7]	M	07/2001	Summer	29.0	4
Cor1	Co1	01 11 08 06	Corsica	*A*. *sylvaticus*	[6], [7]	-	11/2001	Winter	-	-
Cor2	C02	01 11 08 24	Corsica	*A*. *sylvaticus*	[6], [7]	-	11/2001	Winter	-	-
Cor5	Co5	01 11 14 02	Corsica	*A*. *sylvaticus*	[6], [7]	-	11/2001	Winter	-	-

* *Apodemus sylvaticus* is the only *Apodemus* species found on Mediterranean islands [4]: Corsica, Menorca, Mallorca, Sicily, Porquerolles

*Apodemus sylvaticus* and *Apodemus flavicollis* can be found in sympatry in Py and Calabria [5], [4]
