# Supplementary material for: What Do Pneumocystis Organisms Tell Us about the Phylogeography of Their Hosts? The Case of the Woodmouse Apodemus sylvaticus in Continental Europe and Western Mediterranean Islands
Source: PLoS One. 2015 Apr 1;10(4):e0120839. doi: 10.1371/journal.pone.0120839 (PMC4382281; doi:10.1371/journal.pone.0120839)
Supplement: S1 Table — (DOCX) [file pone.0120839.s001.docx]

**S1_ Table:** Divergence matrix of *Pneumocystis* combined mtLSUrDNA and mtSSUrDNA sequences amplified from 30 woodmice, two rodents (*P. murina* from mouse *Mus musculus* and *Pneumocystis* from squirrel *Sciureus aestuans*).

|  | **% of divergence from** | | | | | | | | | | | | | | | | | | | | | | | | | | | | | | |
| --- | --- | --- | --- | --- | --- | --- | --- | --- | --- | --- | --- | --- | --- | --- | --- | --- | --- | --- | --- | --- | --- | --- | --- | --- | --- | --- | --- | --- | --- | --- | --- |
| **Woodmice samples or *Pneumocystis* species** | 1 | 2 | 3 | 4 | 5 | 6 | 7 | 8 | 9 | 10 | 11 | 12 | 13 | 14 | 15 | 16 | 17 | 18 | 19 | 20 | 21 | 22 | 23 | 24 | 25 | 26 | 27 | 28 | 29 | 30 | 31 |
| 1. Sc12 |  |  |  |  |  |  |  |  |  |  |  |  |  |  |  |  |  |  |  |  |  |  |  |  |  |  |  |  |  |  |  |
| 2. Sc13 | 00.96 |  |  |  |  |  |  |  |  |  |  |  |  |  |  |  |  |  |  |  |  |  |  |  |  |  |  |  |  |  |  |
| 3. Sc3 | 00.97 | 00.39 |  |  |  |  |  |  |  |  |  |  |  |  |  |  |  |  |  |  |  |  |  |  |  |  |  |  |  |  |  |
| 4. Sc5 | 00.77 | 00.96 | 00.97 |  |  |  |  |  |  |  |  |  |  |  |  |  |  |  |  |  |  |  |  |  |  |  |  |  |  |  |  |
| 5. Sc8 | 01.35 | 00.38 | 00.78 | 00.58 |  |  |  |  |  |  |  |  |  |  |  |  |  |  |  |  |  |  |  |  |  |  |  |  |  |  |  |
| 6. Sc9 | 01.35 | 00.39 | 00.77 | 00.58 | 00.00 |  |  |  |  |  |  |  |  |  |  |  |  |  |  |  |  |  |  |  |  |  |  |  |  |  |  |
| 7. Sc14 | 00.77 | 00.96 | 00.98 | 00.00 | 00.58 | 00.58 |  |  |  |  |  |  |  |  |  |  |  |  |  |  |  |  |  |  |  |  |  |  |  |  |  |
| 8. Sc16 | 00.96 | 00.77 | 01.16 | 00.96 | 00.39 | 00.38 | 00.96 |  |  |  |  |  |  |  |  |  |  |  |  |  |  |  |  |  |  |  |  |  |  |  |  |
| 9. Cor1 | 00.58 | 01.16 | 01.16 | 00.97 | 01.54 | 01.54 | 00.97 | 01.54 |  |  |  |  |  |  |  |  |  |  |  |  |  |  |  |  |  |  |  |  |  |  |  |
| 10. Cor2 | 00.58 | 01.15 | 01.16 | 00.19 | 00.77 | 00.77 | 00.20 | 00.77 | 00.76 |  |  |  |  |  |  |  |  |  |  |  |  |  |  |  |  |  |  |  |  |  |  |
| 11. Cor5/cal39 | 00.96 | 01.54 | 01.54 | 00.58 | 01.15 | 01.15 | 00.58 | 01.16 | 00.38 | 00.38 |  |  |  |  |  |  |  |  |  |  |  |  |  |  |  |  |  |  |  |  |  |
| 12. Cal6 | 01.54 | 01.34 | 01.35 | 00.77 | 00.96 | 00.96 | 00.77 | 01.35 | 01.92 | 01.15 | 01.53 |  |  |  |  |  |  |  |  |  |  |  |  |  |  |  |  |  |  |  |  |
| 13. Cal7 | 00.77 | 01.35 | 01.35 | 00.39 | 00.96 | 00.95 | 00.39 | 00.96 | 00.96 | 00.19 | 00.57 | 01.34 |  |  |  |  |  |  |  |  |  |  |  |  |  |  |  |  |  |  |  |
| 14. Cal8 | 00.58 | 01.15 | 01.16 | 00.19 | 00.77 | 00.77 | 00.20 | 00.77 | 00.77 | 00.00 | 00.38 | 01.15 | 00.19 |  |  |  |  |  |  |  |  |  |  |  |  |  |  |  |  |  |  |
| 15. Spa4 | 01.35 | 01.55 | 01.55 | 01.35 | 01.93 | 01.93 | 01.35 | 02.32 | 01.55 | 01.54 | 01.93 | 01.54 | 01.74 | 01.54 |  |  |  |  |  |  |  |  |  |  |  |  |  |  |  |  |  |
| 16. Spa15 | 01.35 | 02.32 | 02.31 | 01.35 | 01.93 | 01.92 | 01.35 | 01.54 | 01.93 | 01.16 | 01.54 | 01.54 | 01.35 | 01.16 | 00.77 |  |  |  |  |  |  |  |  |  |  |  |  |  |  |  |  |
| 17. Spa19/Mon5/Por15 | 01.36 | 01.55 | 01.55 | 01.35 | 01.93 | 01.93 | 01.35 | 02.32 | 01.54 | 01.54 | 01.93 | 01.54 | 01.73 | 01.55 | 00.00 | 00.77 |  |  |  |  |  |  |  |  |  |  |  |  |  |  |  |
| 18. Mon6 | 01.81 | 02.01 | 02.02 | 01.83 | 02.43 | 02.43 | 01.83 | 02.84 | 01.61 | 02.03 | 02.02 | 02.04 | 02.23 | 02.03 | 00.40 | 01.22 | 00.40 |  |  |  |  |  |  |  |  |  |  |  |  |  |  |
| 19. Mn58 | 00.96 | 01.93 | 01.93 | 01.73 | 02.31 | 02.30 | 01.73 | 01.92 | 01.54 | 01.53 | 01.92 | 01.92 | 01.72 | 01.54 | 00.39 | 00.38 | 00.38 | 00.80 |  |  |  |  |  |  |  |  |  |  |  |  |  |
| 20. Pyr1 | 01.54 | 02.51 | 02.50 | 01.54 | 02.12 | 02.11 | 01.55 | 01.74 | 02.12 | 01.35 | 01.74 | 01.74 | 01.53 | 01.35 | 00.97 | 00.19 | 00.97 | 01.42 | 00.57 |  |  |  |  |  |  |  |  |  |  |  |  |
| 21. Pyr14 | 01.36 | 01.55 | 01.56 | 01.35 | 01.93 | 01.94 | 01.35 | 02.32 | 01.55 | 01.55 | 01.94 | 01.54 | 01.74 | 01.55 | 00.00 | 00.77 | 00.00 | 00.40 | 00.39 | 00.97 |  |  |  |  |  |  |  |  |  |  |  |
| 22. Pyr3 | 02.53 | 02.72 | 02.71 | 01.75 | 02.33 | 02.33 | 01.75 | 02.72 | 02.72 | 01.94 | 02.33 | 01.94 | 02.13 | 01.94 | 01.17 | 01.17 | 01.17 | 01.64 | 01.55 | 00.97 | 01.17 |  |  |  |  |  |  |  |  |  |  |
| 23. Spa3 | 01.74 | 01.93 | 01.94 | 00.97 | 01.55 | 01.74 | 00.97 | 01.93 | 01.94 | 01.16 | 01.55 | 01.16 | 01.54 | 01.16 | 00.39 | 00.58 | 00.39 | 00.82 | 00.96 | 00.77 | 00.39 | 00.97 |  |  |  |  |  |  |  |  |  |
| 24. Spa5 | 01.95 | 02.15 | 01.96 | 01.18 | 01.77 | 01.77 | 01.17 | 02.16 | 02.16 | 01.37 | 01.77 | 01.38 | 01.57 | 01.37 | 00.58 | 00.58 | 00.58 | 01.05 | 00.97 | 00.78 | 00.58 | 00.99 | 00.19 |  |  |  |  |  |  |  |  |
| 25. Mon4 | 03.67 | 03.86 | 03.67 | 02.90 | 03.48 | 03.47 | 02.89 | 03.87 | 03.87 | 03.10 | 03.49 | 03.11 | 03.28 | 03.10 | 02.32 | 02.31 | 02.32 | 02.46 | 02.68 | 02.50 | 02.32 | 02.71 | 01.93 | 02.17 |  |  |  |  |  |  |  |
| 26. Mon12 | 02.01 | 02.20 | 02.20 | 01.61 | 02.21 | 02.20 | 01.62 | 02.62 | 01.80 | 01.81 | 01.81 | 01.82 | 02.01 | 01.81 | 00.60 | 01.01 | 00.60 | 01.01 | 01.00 | 01.21 | 00.60 | 01.42 | 00.61 | 00.82 | 02.23 |  |  |  |  |  |  |
| 27. Mon23 | 03.10 | 03.29 | 03.10 | 02.71 | 03.29 | 03.29 | 02.71 | 03.68 | 03.29 | 02.90 | 03.29 | 02.91 | 03.10 | 02.91 | 01.74 | 02.12 | 01.74 | 01.65 | 02.12 | 02.32 | 01.74 | 02.53 | 01.74 | 01.97 | 00.97 | 01.42 |  |  |  |  |  |
| 28. Ma47/Ma59/Ma51 | 02.12 | 02.31 | 02.32 | 01.35 | 01.93 | 01.93 | 01.35 | 02.32 | 01.54 | 01.54 | 01.16 | 01.55 | 01.73 | 01.55 | 00.77 | 00.77 | 00.77 | 00.82 | 01.15 | 00.96 | 00.77 | 01.17 | 00.39 | 00.59 | 02.32 | 00.61 | 02.13 |  |  |  |  |
| 29. Ma53/Ma62/Ma66/Ma71 | 01.93 | 02.12 | 02.12 | 01.55 | 02.12 | 02.12 | 01.54 | 02.51 | 01.35 | 01.73 | 01.35 | 01.74 | 01.93 | 01.74 | 00.58 | 00.96 | 00.58 | 00.61 | 00.96 | 01.16 | 00.58 | 01.36 | 00.58 | 00.78 | 02.51 | 00.40 | 01.93 | 00.19 |  |  |  |
| 30. Mn33 | 01.74 | 01.93 | 01.94 | 00.97 | 01.54 | 01.54 | 00.96 | 01.93 | 01.93 | 01.16 | 01.54 | 01.16 | 01.35 | 01.16 | 00.39 | 00.39 | 00.39 | 00.82 | 00.76 | 00.58 | 00.39 | 00.78 | 00.19 | 00.19 | 01.93 | 00.61 | 01.74 | 00.39 | 00.58 |  |  |
| 31. *P.* sp. from a squirrel | 15.92 | 15.17 | 15.54 | 15.35 | 15.11 | 15.08 | 15.34 | 15.52 | 15.35 | 15.53 | 15.32 | 15.36 | 15.71 | 15.56 | 14.37 | 14.86 | 14.34 | 14.29 | 14.88 | 15.06 | 14.19 | 14.97 | 14.35 | 14.83 | 15.84 | 14.44 | 15.63 | 14.10 | 14.12 | 14.31 |  |
| 32. *P. muris* | 08.29 | 08.29 | 08.07 | 07.87 | 08.27 | 08.28 | 08.25 | 08.71 | 08.06 | 08.06 | 08.06 | 07.64 | 08.28 | 08.07 | 07.21 | 07.63 | 07.20 | 07.39 | 07.62 | 07.84 | 07.22 | 08.08 | 07.22 | 07.54 | 08.50 | 07.12 | 07.63 | 07.20 | 07.20 | 07.20 | 15.30 |

Numbered column headings (1 to 32) correspond to numbered sources of host species or woodmice DNA in the first column. The calculation of the divergence matrix does not take into account sites with an indel (gap). Thus, when sequences show 0.00 % of divergence, it cannot be stated that sequences are identical.
