# Supplementary figures and images for: What Do Pneumocystis Organisms Tell Us about the Phylogeography of Their Hosts? The Case of the Woodmouse Apodemus sylvaticus in Continental Europe and Western Mediterranean Islands
Source: PLoS One. 2015 Apr 1;10(4):e0120839. doi: 10.1371/journal.pone.0120839 (PMC4382281; doi:10.1371/journal.pone.0120839)

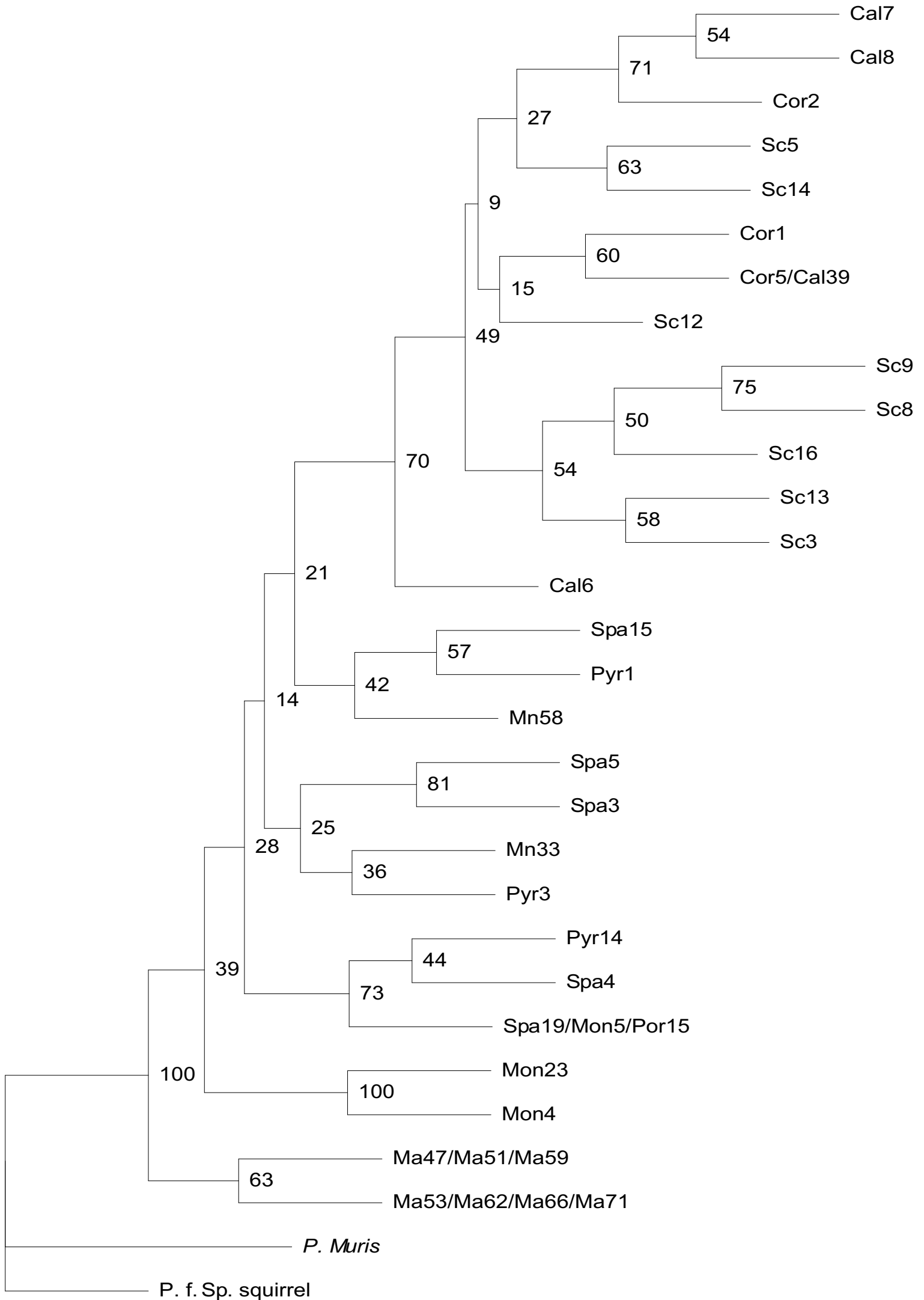

Supplement: S2 Fig — NJ is inferred from the combined mitochondrial small subunit (mtSSU) and large subunit (mtLSU) rRNA sequences. The percentages displayed above the branches are the frequencies with which a given branch appeared in 1,000 bootstrap replications. (PDF) [file pone.0120839.s003.pdf]

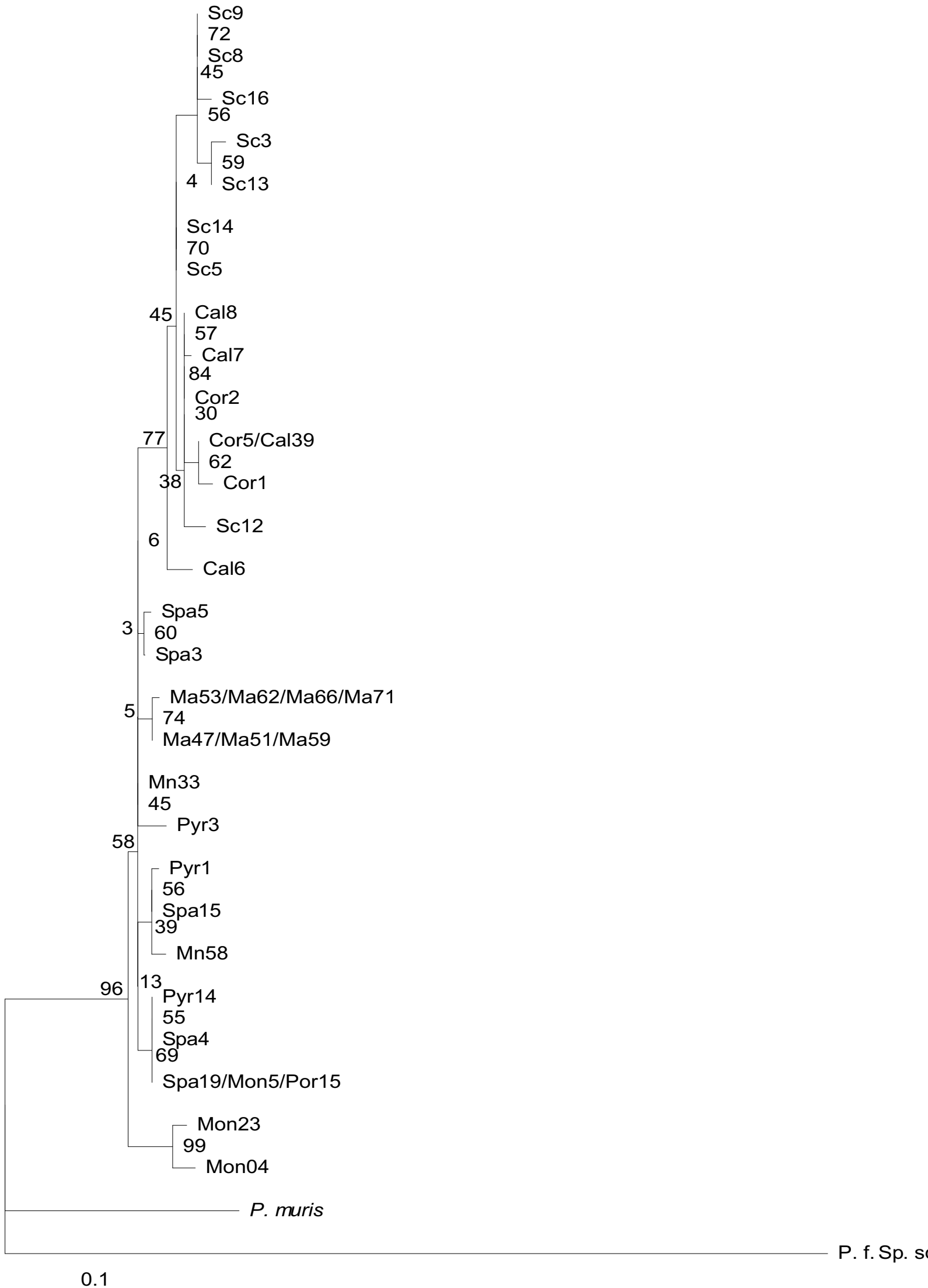

Supplement: S3 Fig — ML is inferred from the combined mitochondrial small subunit (mtSSU) and large subunit (mtLSU) rRNA sequences. The percentages displayed above the branches are the frequencies with which a given branch appeared in 1,000 bootstrap replications. (PDF) [file pone.0120839.s004.pdf]

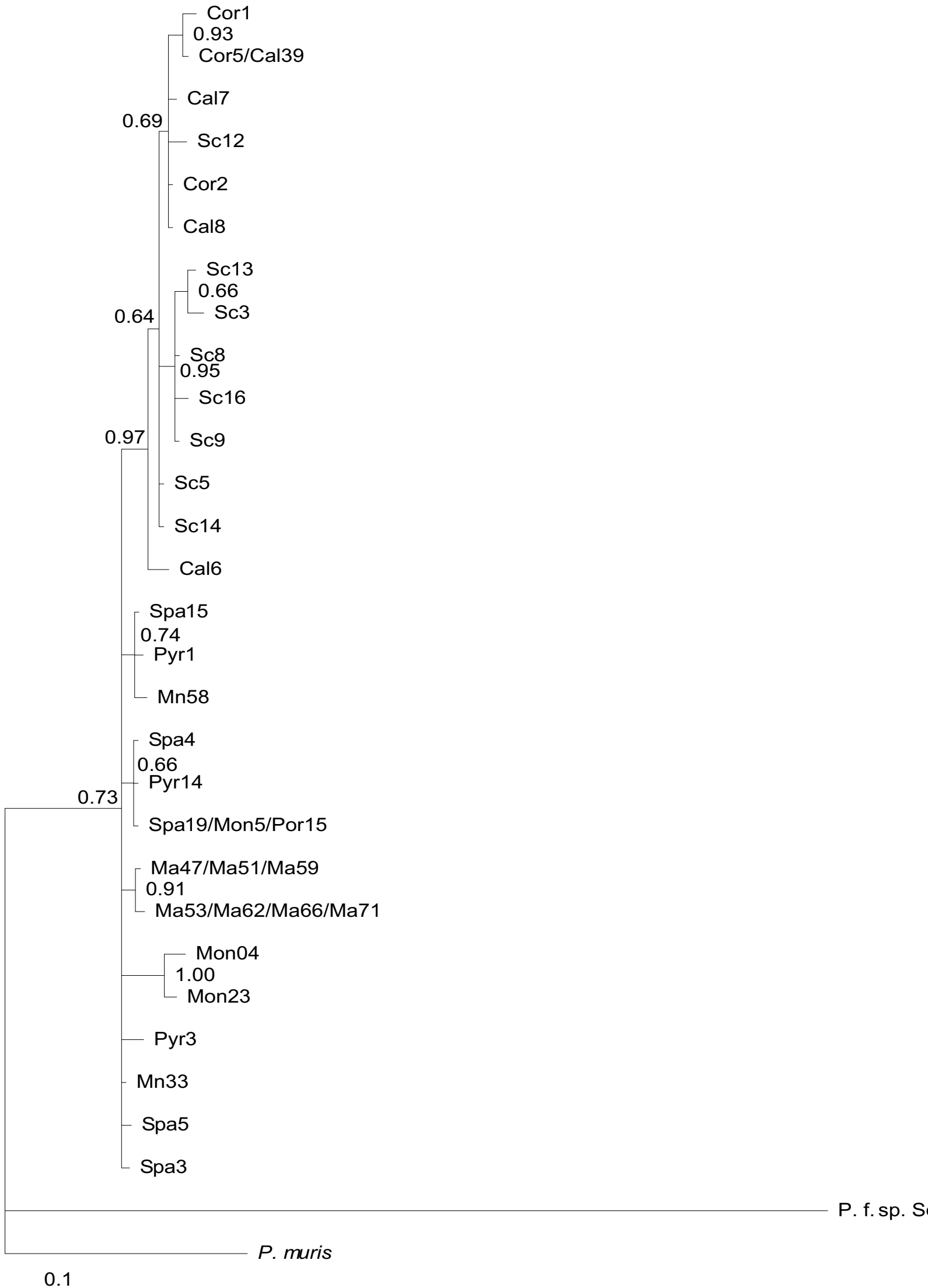

Supplement: S4 Fig — MB are inferred from the combined mitochondrial small subunit (mtSSU) and large subunit (mtLSU) rRNA sequences. The percentages displayed above the branches are the frequencies with which a given branch appeared in 1,000 bootstrap replications. (PDF) [file pone.0120839.s005.pdf]

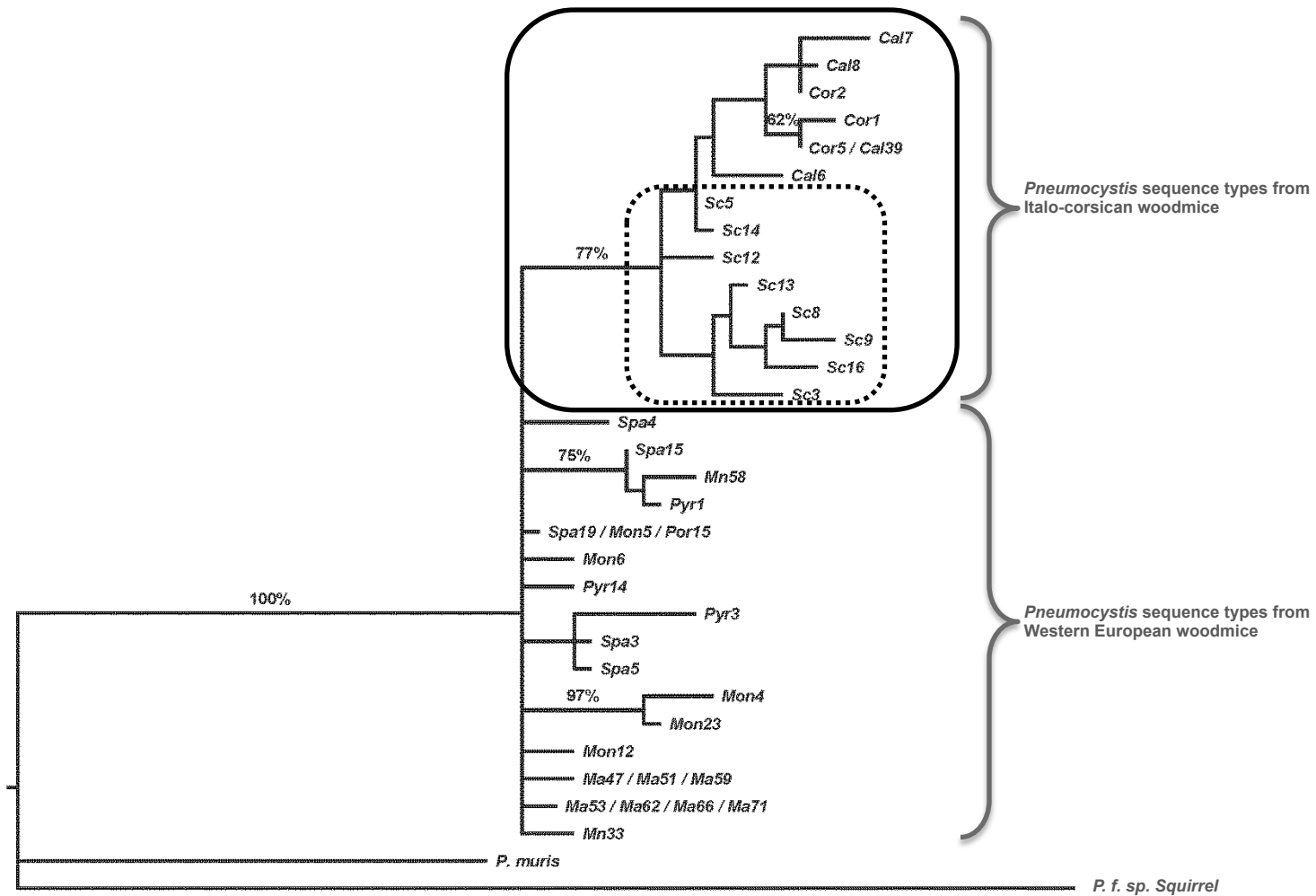

Supplement: S5 Fig — MP is inferred from the combined mitochondrial small subunit (mtSSU) and large subunit (mtLSU) rRNA sequences. The percentages displayed above the branches are the frequencies with which a given branch appeared in 1,000 bootstrap replications. *Bootstrap values below 50% are reported. (PDF) [file pone.0120839.s006.pdf]
